# Analysis of Veterinary Drug and Pesticide Residues Using the Ethyl Acetate Multiclass/Multiresidue Method in Milk by Liquid Chromatography-Tandem Mass Spectrometry

**DOI:** 10.1155/2016/2170165

**Published:** 2016-05-16

**Authors:** Husniye Imamoglu, Elmas Oktem Olgun

**Affiliations:** ^1^Istanbul Sabahattin Zaim University, Halkalı, 34303 Istanbul, Turkey; ^2^TUBITAK Marmara Research Centre, Food Institute, P.O. Box 21, Gebze, 41470 Kocaeli, Turkey

## Abstract

A rapid and simple multiclass, ethyl acetate (EtOAc) multiresidue method based on liquid chromatography coupled with tandem mass spectrometry (LC-MS/MS) detection was developed for the determination and quantification of 26 veterinary drugs and 187 total pesticide residues in milk. Sample preparation was a simple procedure based on liquid–liquid extraction with ethyl acetate containing 0.1% acetic acid, followed by centrifugation and evaporation of the supernatant. The residue was dissolved in ethyl acetate with 0.1% acetic acid and centrifuged prior to LC-MS/MS analysis. Chromatographic separation of analytes was performed on an Inertsil X-Terra C18 column with acetic acid in methanol and water gradient. The repeatability and reproducibility were in the range of 2 to 13% and 6 to 16%, respectively. The average recoveries ranged from 75 to 120% with the RSD (*n* = 18). The developed method was validated according to the criteria set in Commission Decision 2002/657/EC and SANTE/11945/2015. The validated methodology represents a fast and cheap alternative for the simultaneous analysis of veterinary drug and pesticide residues which can be easily extended to other compounds and matrices.

## 1. Introduction

Veterinary drugs are widely used in medical and veterinary practices to treat and prevent disease as well as improve feed efficiency and increase animal growth rates [1]. Pesticides are also widely used to enhance food production by protecting food crops from potentially harmful and destructive pests [2]. However, the resulting occurrence of contaminants and/or residues in the human diet represents an issue of high concern.

According to the European Union, the maximum residue limit (MRL) in dairy milk is 100 *μ*g/kg for tetracycline and sulfenamide, 50 *μ*g/kg for macrolides and quinolones, and 10 *μ*g/kg for pesticides. Sensitive analytic methods have been developed to monitor and detect the MRL values in the dairy milk [3]. There are ultra-high pressure liquid chromatography mass spectrometry (UHPLC-MS/MS) methods reported to detect multiple residues of *β*-lactams [4, 5], as well as pesticides and mycotoxins [6], and some antihelminthic drugs and phenylbutazone [7].

Milk is a complex food that is high in fat and protein, and such ingredients may cause interactions in the analytical processes. Therefore, sample preparation is required, particularly in extraction and cleanup. Formerly, sample preparation methods were based on a few compounds or a single class of such drugs. Applying common extraction procedures and developing chromatographic conditions are difficult in multiclass and multiresidue analyses. Solid phase extraction methods have been applied, after the phases of protein precipitation and centrifugation, in order to observe the fluoroquinolones [8], veterinary drugs [9], mycotoxins, and pesticides in milk [6, 10]. However, these methods are generally found to be time-consuming and require large volumes of organic solvents.

Multiresidue veterinary drugs that were developed for milk tests depend on various extraction and cleanup principles. One of the most accepted approaches is to dilute a sample of milk with a solvent like acetonitrile and then to centrifuge and evaporate the obtained supernatant organic extract [11, 12]. Some multiclass analytical method applications by LC-MS/MS or LC-TOF/MS, related to homogenized or raw milk, that have the ability to specify undesirable chemicals, such as tetracycline, quinolone, sulfonamide, peptide, hormone, nonsteroidal anti-inflammatory anthelmintic drugs, mycotoxin, and pesticides, can be found in the literature [7]. Yet most of these methods are unable to offer satisfactory recovery of a large range of compounds of different polarities [13, 14].

Most methods for the analysis of veterinary residues have some disadvantages, including high solvent consumption, tedious SPE cleanup steps that require extended time for analysis, and high costs. Therefore, these types of methods are not applied for routine analyses. The Quick Easy Cheap Effective Rugged Safe (QuEChERS) methodology, which was originally developed for pesticide analysis, has recently been proposed for the analysis of veterinary drugs using different matrices [15–18]. However, QuEChERS was found to be inconvenient for the recovery of polar veterinary drugs, including penicillin, tetracycline, and quinolone [13, 18, 19]. Therefore, there is still a great need for simple and rapid multiresidue analytical methods for simultaneously determining veterinary drug and pesticide residues in milk.

In this study, we prepared milk samples by using a procedure based on a simple liquid-to-liquid extraction. This method utilized a simple and quick sample preparation procedure using a single extraction step. Through this method, milk samples were analyzed for the determination of both veterinary drugs and pesticide residues by utilizing liquid chromatography-tandem mass spectrometry (LC-MS/MS). As a result, the reduced use of chemicals and steps in the sample preparation phase, together with the avoidance of a sample cleanup step, simplified the sample pretreatment and reduced the overall total cost. Finally, in addition to reducing analyses costs, the method provided a higher recovery of compounds of various polarities and improved the simplicity of detection efforts.

## 2. Materials and Methods

### 2.1. Reagents and Chemicals

HPLC grade acetonitrile (ACN), methanol, ethyl acetate (EtOAc) (Lichrosolv, purity ≥ 99.9), and glacial acetic acid (Emprove, 100%) were purchased from Merck (Darmstadt, Germany). The water used to prepare the solutions was purified in a Milli-Q Plus system (EMD Millipore, Billerica, MA). Magnesium sulfate, sodium chloride, Supelclean*™* primary secondary amine (PSA), pure tetracyclines, sulfonamides, quinolones, macrolides, and antibiotics were provided from Sigma Aldrich (St. Louis, Missouri, USA) and the pesticides were provided from Dr. Ehrenstrorfer (Augsburg, Germany).

### 2.2. Samples

All pasteurized whole milk samples were purchased from local markets. Also, raw milk was used for interference and specificity/selectivity as a blank.

*Standard Solutions*. Individual stock solutions of the veterinary drugs and pesticides were prepared in acetonitrile at a concentration of 1000 mg/kg. A mixed intermediate standard solution was prepared by diluting the stock standard solutions of the veterinary drugs and pesticides in acetonitrile at a concentration of 10 mg/kg. Stock and intermediate standard solutions were stored at 4°C in amber flasks and were found stable for at least 6 months.

### 2.3. Extraction Procedures

#### 2.3.1. Ethyl Acetate Extraction without Salting Procedure

Milk samples, upon arrival at our laboratory, were kept at refrigerator temperature (10 ± 4°C) until analysis. For the preparation an aliquot of approximately 5 mL milk sample was pipetted in a 50 mL polypropylene centrifuge tube. Then, 200 mcL acetic acid was added to 10 mL of ethyl acetate. After vortex for 3 minutes, the mixture was centrifuged at 5000 rpm for 10 minutes. The upper phase was taken in 15 mL centrifuge tube and was dried under a gentle stream of nitrogen, and the residue was reconstituted with 1000 mcL of mobile phase A/mobile phase B (80/20). The sample was vortexed vigorously for 10 minutes. The extract was filtered through a 0.45 *μ*m filter prior to LC-MS/MS analysis.

#### 2.3.2. Acetonitrile Extraction without Salting Procedure

Approximately 5 mL milk sample was pipetted in a 50 mL polypropylene centrifuge tube. Then, 10 mL of acetonitrile and 200 mcL acetic acid were added to milk. After mixing by a vortex stirrer for 3 minutes, the mixture was centrifuged at 5000 rpm for 10 minutes. The upper phase was taken in 15 mL centrifuge tube and was dried under a gentle stream of nitrogen, and the residue was reconstituted with 1000 mcL of mobile phase A/mobile phase B (80/20). The sample was vortexed vigorously for 10 minutes. The extract was filtered through a 0.45 *μ*m filter prior to LC-MS/MS analysis.

#### 2.3.3. QuEChERS Extraction Procedure

Approximately 5 mL milk sample was pipetted in a 50 mL polypropylene centrifuge tube. Then, 2 g of magnesium sulfate and 1 g of sodium acetate were added to milk samples [15]. Then, 10 mL of acetonitrile and 100 mcL acetic acid were added to milk samples. After vortex for 3 minutes, the mixture was centrifuged at 5000 rpm for 10 minutes. The upper phase was taken in 15 mL centrifuge tube and was dried under a gentle stream of nitrogen, and the residue was reconstituted with 1000 mcL of mobile phase A/mobile phase B (80/20). The extract was transferred to a 2 mL Eppendorf microtube containing 50 mg PSA and 200 mg magnesium sulfate. Then, the tube was centrifuged at 4000 rpm during 5 minutes. The extract was filtered through a 0.45 *μ*m filter prior to LC-MS/MS analysis.

### 2.4. LC-MS/MS Analysis

The chromatographic analyses were performed using an HPLC system consisting of a binary pump (Shimadzu UFLC LC-20AD model), Shimadzu automatic injector (Autosampler SIL-20A HT model), and a column oven (CTO-20AC). Analytical columns, Symmetry® C18 2.1 × 150 mm id, 5 *μ*m particle size (Waters, Milford, MA), and Waters XTerra C18 150 mm × 2.1 mm id, 5 *μ*m particle size (Waters, Milford, MA), were tested. Chromatographic separation of veterinary drugs and pesticides was carried out on a Waters Symmetry C18 column. The method used a gradient mobile phase containing 0.1% acetic acid water and mobile phase B containing methanol. The column temperature was maintained at 40°C with a flow rate of 0.3 mL/min. The gradient profile was scheduled as follows: initial proportion (98% A and 2% B) for 0.3 minutes, linear increase to 80% (B) until 7 minutes, and hold of 80% (B) for 3 minutes. The injection volume was 50 *μ*L. The chromatographic system was coupled to electrospray ionization (ESI) source followed by an Applied Biosystems MDS SCIEX 4500 Q TRAP mass spectrometer. The MS/MS detector conditions were as follows: curtain gas 20 mL/min, exit potential 10 V, ion source gas 1 and ion source gas 2 set at 50 mL/min, ion spray voltage 5500 V, and turbo spray temperature set at 550°C. MS data were acquired in the positive ion ESI mode using two alternating MS/MS scan events. Two transitions were monitored for each analyte. The selected molecular ion and optimized collision voltages of product ions used for quantification, confirmation, and ion ratio were summarized in Table 1. Applied Biosystems SCIEX Analyst software version 1.6 was employed for data acquisition and processing. Quantification was by comparison with a six-point calibration (0.0, 0.01, 0.025, 0.05, 0.1, and 0.2 mg/kg) in matrix-matched calibration.

### 2.5. Validation Study

The analytical method developed for determination of veterinary drug and pesticide residues in milk was validated according to EU Decision 2002/657/EC [16] and SANTE/11945/2015 [17]. The following parameters were evaluated in the validation procedure: selectivity, sensitivity, linearity, precision (intraday and interday reproducibility), accuracy and CC*α* and CC*β*, LOD, and LOQ.

## 3. Results and Discussion

### 3.1. Optimization of the Extraction Procedures

Ethyl acetate extraction without salt procedure was chosen to be performed in this study because of its advantages. There was no need to use salt and it could give lower detection limit in terms of volatile characteristic of ethyl acetate.

**Figure 1:**
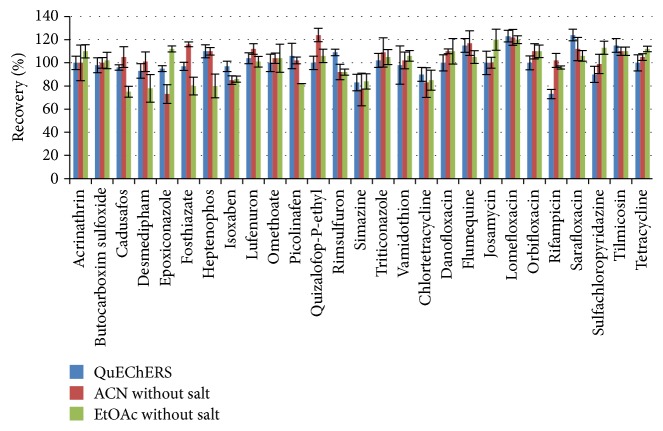
Recovery data for three different extraction procedures.

Recovery values showed no difference among three different extraction procedures (acetonitrile extraction, QuEChERS extraction, and ethyl acetate extraction without salting procedure) (Figure 1).

**Figure 2:**
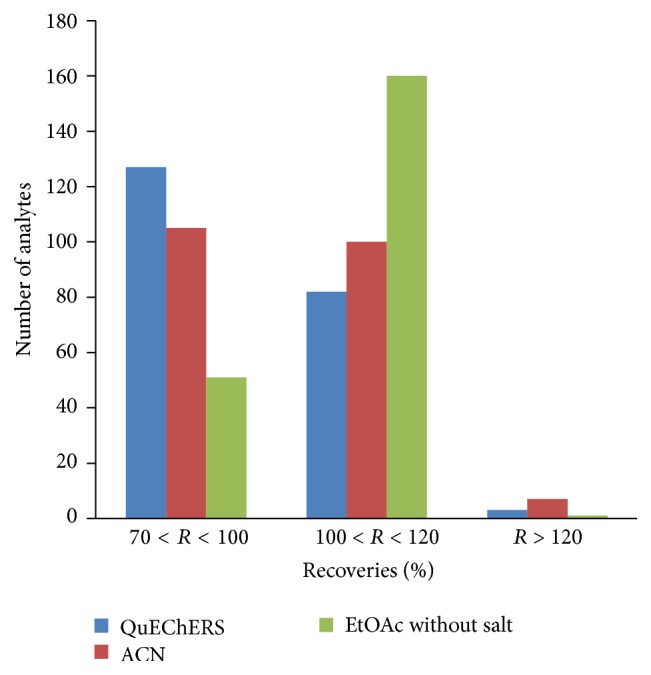
Recovery (%) data obtained using extraction procedures; QuEChERS, ACN, and EtOAc without salt.

The recovery values expressed as recovery % are all within the reference range of 70–120%. Comparing three procedures, EtOAc without salt provided recoveries between 100% and 120% for a higher number of veterinary drugs and pesticides (26 veterinary drugs and 134 pesticides; total of 160 compounds) than QuEChERS (82 compounds) and ACN (100 compounds), as it can be observed in Figure 2. In terms of extraction recoveries, EtOAc was found to be a suitable extraction procedure for all 26 veterinary drugs and most of the pesticides analyzed in this study. Only one analyte (propham) showed *R* > 120 for EtOAc.

Accuracy was evaluated in terms of relative standard deviation (RSD) by spiking blank samples with the corresponding volume of the multicompound working standard solution. RSD was evaluated at 50 *μ*g/kg by spiking six blank samples at each level for three procedures that provided similar RSD values. These values were within 1 < RSD < 10 for 75% of each analyte in the three procedures. These results indicated that the EtOAc without salt method was precise, accurate, and reliable for the analysis of the veterinary drug and pesticide compounds in the milk samples as an alternative method.

### 3.2. LC-MS/MS

Mobile phase was acidified with acetic acid in methanol and water. Also, study [18] in the literature was performed for the comparison. Formic acid in acetonitrile and water was used as a mobile phase in [18]. According to analyte intensities, our results gave better peak shapes than chromatograms in [18]. The dried residue was redissolved in a mixture of MeOH/water with 0.1% acetic acid to test different reconstitution solvents. This composition produced better peak shapes for all analytes compared with water-methanol (80 : 20) that gave lower response. Increasing acetic acid to 1% in the mixture did not improve chromatography but caused extra peaks in the background noise.

### 3.3. Validation Study

#### 3.3.1. Selectivity

The selectivity of the method was assessed by duplicate analysis of 10 blank milk samples. No peaks of interfering compounds were observed within the intervals of the retention time of the analytes in any of these samples.

#### 3.3.2. Linearity

Linearity was evaluated from the calibration curves by triplicate analyses of blank milk samples fortified with the analytes at six (0.0, 0.01, 0.025, 0.05, 0.1, and 0.2 mg/kg) concentration levels. Linearity was expressed as the coefficient of linear correlation (*r*) and from the slope of the calibration curve. The linearity of the analytical response across the studied range was excellent, with correlation coefficients higher than 0.997 for all analytes, which was similar to the findings in [19]. The authors [20] found correlation coefficients higher than 0.992 for all analytes, which was a lower score than ours.

#### 3.3.3. Decision Limit and Detection Capability

CC*α* is defined as the limit at and above which it can be concluded with an error probability of *α* that a sample is noncompliant. CC*β* is defined as the smallest content of the substance that may be detected, identified, and/or quantified in a sample with an error probability of *β*. The CC*α* and CC*β* were determined by analysis of 10 blank milk samples and the signal-to-noise (S/N) ratio is calculated at the time window in which the analyte is expected. The CC*α* values were calculated as three times the S/N ratio. The CC*β* was calculated by analyzing 10 blank samples spiked with concentration at CC*α*. Then the CC*α* value was added up to 1.64 times the corresponding standard deviation. Then, a preliminary experiment was conducted to check if all compounds were detected when spiked at their CC*α* level (Table 2).

**Figure 3:**
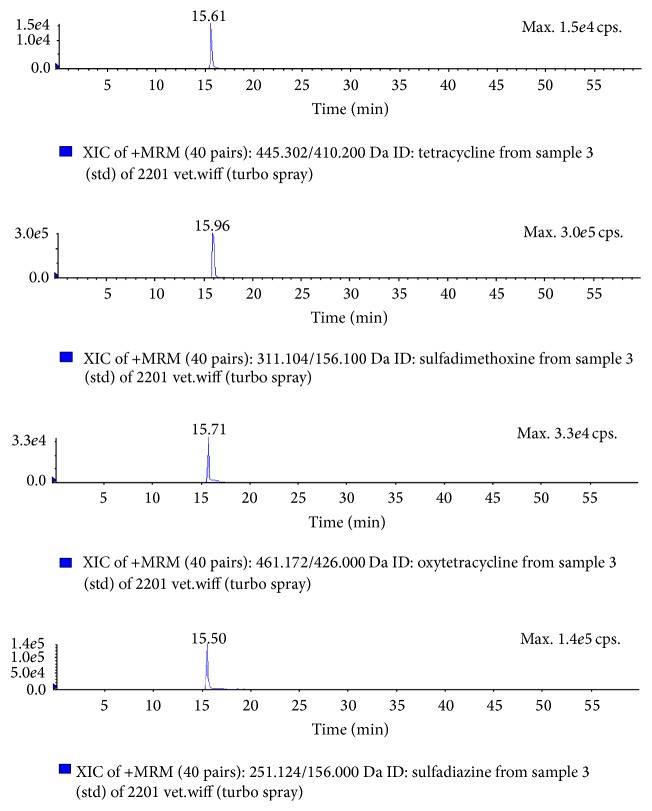
MRM chromatograms of milk samples at the LOQ level of tetracyclines, sulfadimethoxine, oxytetracycline, and sulfadiazine (10 *μ*g/kg).

In Figure 3, very satisfactory S/N ratios were obtained for all analytes at LOQ level. The lowest LOQ value was 50 *μ*g/kg for tetracyclines and for sulfonamides 20 *μ*g/kg in veterinary drugs in [20] while it was 10 *μ*g/kg for both of them in our study except ciprofloxacin and quinolone. Figure 3 shows MRM chromatograms of milk samples at the lowest validation concentration at LOQ level.

#### 3.3.4. Accuracy and Precision

The accuracy was evaluated by recovery tests, analyzing fortified blank samples at the same concentration levels used in the precision tests (0.01, 0.025, and 0.05 mg/kg). The accuracy and precision of the method results (Table 2) confirmed the values given in Decision 2002/657/EC [16]. Thus, the mean accuracy values obtained in the recovery tests were between 61 and 130%. The precision of the method was determined in two stages: repeatability (intraday) and intermediate precision (interday). Repeatability was expressed by the RSD of the results from six replicates analyzed on the same day by the same analyst using the same instrument. The intermediate precision was expressed by the RSD of the results of eighteen analyses performed on three different days (*n* = 3), six analyses/day, by the same analyst using the same instrument. The relative standard deviation (RSD) of interday values of veterinary drugs and pesticides analyzed by the present method was 2 to 13% and for the intraday test 5–19% (Table 2), while relative standard deviation (RSDr) of intraday values was 4–26% in [20].

#### 3.3.5. Matrix Effects

Evaluation of matrix effect is important during validation of analytical methods using the LC-MS/MS technique. The ionization efficiency of the analytes in ESI source may be affected by matrix interference. In order to evaluate the degree of ion suppression or signal enhancement, calibration curves were established with and without matrix. Matrix-induced effects were assessed by comparing the slopes of these calibration curves using the following formula: matrix effect (ME) = 1 − (*a*_matrix_/*a*_standard_) × 100, where *a*_matrix_ and *a*_standard_ are the slopes of calibration straight lines for standard and matrix-matched calibration graphs. The matrix-matched calibration curves were constructed using milk samples (5 g/mL matrix equivalent) prepared in MeOH-water solution with 0.1% acetic acid and spiked with veterinary drug and pesticides at concentration levels of 0.01, 0.025, and 0.05 mg/kg. Matrix effect was further evaluated for ion suppression between the standards prepared in pure solvent and standards prepared in matrix and the matrix effect was found to be in a range of 15–25%. These results showed that standard calibration which was simpler and less time-consuming compared with matrix-matched calibration can effectively be used for quantitation of veterinary drug and pesticides in milk (Table 2).

### 3.4. Real Samples

The method used analyzed more than 220 milk samples submitted to the laboratory for veterinary drug and pesticide residues by the local markets. Two transition ion pairs were monitored for each of the analytes and the ion ratios of detected samples were compared well with those of standards. Retention times of analytes were also confirmed by addition of known standards in detected samples. Eight samples out of 220 milk samples were found to contain residues of veterinary drug and pesticide residues (4% incidence was positive). Sulfadiazine (veterinary drug) residue amount was found between 0.075 and 0.125 mg/L in 2 samples and tetracycline (veterinary drug) amount was found to be 0.015–0.100 mg/L in 4 samples. Carbaryl (pesticide) residue concentration level was 0.005–0.025 mg/L in 2 samples.

## 4. Conclusions

A multiclass/multiresidue procedure with LC-MS/MS detection has been developed and validated to determine and quantify veterinary and pesticide residues in milk. A simple sample preparation method involved liquid extraction salting out procedures in ethyl acetate system, without cleanup steps, and shortening the sample preparation time. Validation of the method was performed according to Commission Decision 2002/657/EC. The method was characterized by good results in terms of recovery, reproducibility, and repeatability allowing the detection of veterinary drug and pesticide residues below the recommended analytical level. Based on these results, LC-MS/MS method with ethyl acetate extraction showed the suitability for sensitive quantification of veterinary and pesticide residues in milk samples for food safety applications. The validated method was applied on 220 real commercial samples. This short protocol can be applicable to a large number of samples for routine analysis and rapid detection.

## Figures and Tables

**Table 1:** LC-MS/MS ion parameters.

Compounds	Precursor ion	Transition 1	Transition 2	Ion ratio
	(*m*/*z*)	(*m*/*z*)	(*m*/*z*)	(%)
2,4-D (negative)	219	160	125	95
2,4,5-T	253	195	197	98
2,4-Dimethylaniline	122	107		80
Acetamiprid	223	126	73	22
Acrinathrin	560	208	181	75
Alachlor	238	162	238	12
Amitraz	294	163	122	75
Atrazine	216	174	104	45
Azoxystrobin	404	372	344	33
Bentazon	(−)239	132	197	78
Bifenazate	(−)300	253	239	79
Bitertanol	339	70	269	81
Boscalid	344	307	140	61
Bromacil	(−)259	205	203	55
Bromuconazole	378	159	70	66
Bromoxynil	(−)274	79	81	67
Bupirimate	317	108	166	86
Buprofezin	307	116	201	93
Butocarboxim sulfoxide	207	75	132	88
Cadusafos	272	159	97	98
Carbaryl	202	145	127	34
Carbendazim	192	160	132	17
Carbofuran	222	165	123	98
Carbosulfan	381	118	160	90
Carboxin	234	143	87	85
Dimethoate	230	199	125	97
Dimethomorph	388	301	165	58
Dimoxystrobin	328	116	205	99
Diniconazole	326	70	159	65
Dinobuton	327	215	152	66
Dinocap (sum)	295	193	209	89
Dinoterb	(−)239	207	176	85
Diphenylamine	171	93	152	17
Disulfoton-sulfoxide	291	185	213	87
Dithianon	(−)296	263	238	86
Diuron	233	72	160	85
Epoxiconazole	330	121	101	80
EPTC	191	128	86	40
Ethiofencarb	226	107	164	81
Ethion	402	385	199	76
Ethirimol	211	98	140	87
Ethofumesate	304	121	161	25
Etoxazole	361	141	113	86
Ethoxyquin	219	160	174	84
Famoxadone	392	238	331	84
Fenamidone	313	92	236	83
Fenamiphos	304	217	202	59
Fenarimol	331	268	81	18
Fenazaquin	307	161	147	80
Fenbuconazole	338	70	125	88
Fenhexamid	303	97	55	63
Fenitrothion	278	125	109	60
Fenoxycarb	302	88	116	25
Fenpropathrin	367	125	350	33
MCPP	(−)213	141	143	18
Metalaxyl-M	280	160	220	85
Mepanipyrim	225	106	77	17
Mesosulfuron-methyl	505	182	83	15
Metazachlor	279	210	134	82
S-Metolachlor	284	252	254	81
Metosulam	419	175	140	94
Metribuzin	215	187	84	29
Monocrotophos	224	127	98	9
Monolinuron	216	126	148	43
Monuron	199	72	126	76
Omethoate	215	125	125	10
Oxadiargyl	341	223	151	87
Oxadiazon	363	220	177	88
Oxadixyl	280	219	133	79
Oxamyl	237	72	90	65
Oxasulfuron	408	150	107	89
Oxycarboxin	269	175	147	31
Oxyfluorfen	362	316	237	27
Penconazole	284	70	159	67
Pendimethalin	282	212	194	19
Pethoxamid	297	131	250	62
Phosalone	368	182	111	31
Phenmedipham	301	136	168	54
Phenthoate	321	163	79	18
Phosmet	318	160	133	13
Phosphamidon	300	127	174	28
Picloram	(−)239	196	123	56
Terbuthylazine	230	174	104	55
Pirimicarb	239	72		93
Thiacloprid	254	126	186	18
Thiamethoxam	292	211	181	39
Thifensulfuron-methyl	389	167	205	14
Thiodicarb	355	88	108	22
Thiophanate-methyl	343	151	192	34
Triadimefon	294	197	225	30
Triadimenol	296	227	70	9
Triallate	304	86	143	67
Triasulfuron	403	167	141	67
Triazophos	314	119	162	54
Tribenuron-methyl	397	155	181	66
Tributylphosphate	268	98		67
Trichlorfon	274	109	221	75
Tridemorph	298	130	116	84
Trifloxystrobin	410	186	206	37
Triflumizole	346	73	278	26
Triticonazole	318	70	125	95
Vamidothion	288	146	118	33
Zoxamide	336	159	189	26
Ciprofloxacin	332	314	288	82
Clindamycin	425	126	82	97
Chlortetracycline	479	462	444	88
Danofloxacin	360	316	342	88
Difloxacin	400	356	299	90
Doxycycline hydrate	445	428	410	97
Flumequine	860	174	109	56
Josamycin	828	109	174	75
Clofentezine	303	138	102	88
Chloridazon	223	104	92	54
Chlorfenvinphos	359	155	99	51
Chlorfluazuron	(−)538	518	355	88
Chloroxuron	292	72	218	87
Chlorpyrifos	350	198	200	8
Chlorsulfuron	359	141	167	89
Chlorthiamid	206	189	154	25
Cinidon-ethyl	412	348	107	26
Cyazofamid	326	108	261	35
Cyclanilide	(−)272	160	228	45
Cycloate	216	154	134	48
Cymoxanil	199	128	111	59
Cyproconazole	292	70	125	65
Cyprodinil	226	93	77	80
Demeton-S-methyl	231	89	61	62
Demeton-S-methylsulfoxide	247	109	169	26
Desmedipham	318	182	136	88
Diallate	271	86	109	36
Diazinon	305	169	97	62
Dichlofluanid	350	123	224	16
Dichlorprop	(−)233	161	125	87
Dichlorvos	221	109	127	15
Difenoconazole	406	251	337	32
Dimethenamid (sum)	277	244	168	68
Fenthion	279	169	247	33
Flazasulfuron	409	182	227	46
Fludioxonil	(−)247	126	169	57
Fluazifop-P-butyl	385	282	328	62
Flufenacet	365	194	152	61
Flufenoxuron	(−)488	156	304	99
Flurochloridone	313	292	145	48
Flurtamone	335	178	247	79
Flusilazole	317	165	247	78
Flutolanil	325	262	242	45
Foramsulfuron	454	182	139	54
Fosthiazate	285	104	228	55
Furathiocarb	384	195	252	62
Heptenophos	251	127	109	9
Hexythiazox	353	228	168	81
Imazalil	297	159	201	88
Imazamox	(−)304	259	217	10
Imazaquin	313	199	128	25
Imazosulfuron	413	156	260	38
Imidacloprid	256	209	175	84
Indoxacarb	529	203	56	84
Ioxynil	(−)370	127	243	99
Iprovalicarb	322	119	203	95
Isazofos	314	120	162	34
Isoproturon	208	72	165	99
Isoxaben	334	165	150	48
Lufenuron	(−)509	326	339	46
Malathion	331	127	99	86
MCPA	(−)199	141	155	94
Picolinafen	378	238	145	57
Mecarbam	331	227	97	96
Pirimiphos-methyl	306	108	164	68
Prochloraz	376	308	266	33
Profenofos	373	303	97	60
Prometryn	242	158	68	68
Propamocarb	190	102	144	39
Propanil	218	162	127	66
Propargite	368	175	231	65
Propham	180	138	120	28
Propiconazole	342	159	69	62
Propyzamide	256	190	173	63
Pymetrozine	219	105	79	11
Pyraclostrobin	389	194	163	98
Pyridaben	365	309	147	78
Pyridaphenthion	341	205	189	88
Pyridate	380	207	351	78
Pyriproxyfen	322	96	185	62
Quinalphos	300	147	163	54
Quinoxyfen	309	197	162	97
Quizalofop-P-ethyl	374	299		56
Rimsulfuron	433	182	325	55
Simazine	202	124	132	70
Spiroxamine	299	144	100	87
Sulfosulfuron	472	211	261	89
Tebuconazole	308	70	125	55
TEPP	291	179	99	96
Terbutryn	242	186	68	51
Tetrachlorvinphos	367	127	241	95
Thiabendazole	203	175	131	12
Oxytetracycline	461	426	443	97
Rifampicin	823	791	151	91
Sarafloxacin	386	368	342	92
Sulfachloropyridazine	285	156	207	75
Sulfaquinoxaline	301	156	108	99
Sulfadiazine	251	156	92	86
Sulfamerazine	265	156	108	89
Sulfathiazole	256	156	92	88
Sulfamethazine	279	186	124	99
Sulfadoxine	311	156	108	97
Sulfapyridine	250	184	156	96
Sulfaclozine	285	156	108	95
Sulfamethoxazole	254	92	108	99
Tilmicosin	869	522	678	97
Tetracycline	445	428	410	85
Lomefloxacin	407	126	82	88
Orbifloxacin	396	352	295	88
Oxolinic acid	262	244	202	89

**Table 2:** Validation results of the developed method.

Compounds	*r* ^2^	LOQ	MRL	CC*α*	CC*β*	% recovery	% RSD	% recovery	% RSD	% recovery	% RSD	% RSDr	Relative uncertainty %	Matrix effect
		(*µ*g/kg)	(*µ*g/kg)	(*µ*g/kg)	(*µ*g/kg)	10 (*µ*g/kg)	10 (*µ*g/kg)	25 (*µ*g/kg)	25 (*µ*g/kg)	50 (*µ*g/kg)	50 (*µ*g/kg)			
2,4-D (negative)	0.997	11	10	21	33	112	8	90	6	108	5	13	32	0.81
2,4,5-T	0.998	9	10	18	26	85	18	102	17	93	9	11	23	0.92
2,4-Dimethylaniline	0.998	8	10	18	26	79	11	110	8	97	8	11	35	0.99
Acetamiprid	0.997	10	10	18	26	97	9	102	8	96	7	11	33	0.92
Acrinathrin	0.998	11	10	18	26	106	10	88	8	104	7	11	35	0.81
Alachlor	0.997	11	10	20	30	108	11	86	4	92	3	13	28	0.81
Amitraz	0.997	9	10	20	30	86	8	76	4	76	3	14	28	0.81
Atrazine	0.997	11	10	17	23	109	6	94	5	91	3	9	30	0.85
Azoxystrobin	0.998	11	10	21	33	114	9	102	5	110	4	14	30	0.92
Bentazon	0.998	12	10	17	23	118	5	94	4	94	4	8	28	0.85
Bifenazate	0.998	11	10	20	30	109	10	102	7	111	6	11	33	0.92
Bitertanol	0.998	11	10	17	23	112	5	98	3	97	3	8	26	0.88
Boscalid	0.998	12	10	15	20	115	6	110	6	101	5	7	30	0.99
Bromacil	0.998	9	10	20	30	91	16	108	14	91	11	13	42	0.97
Bromuconazole	0.997	11	10	20	30	109	9	100	8	95	6	14	30	0.90
Bromoxynil	0.997	10	10	17	23	99	14	106	9	99	6	9	33	0.96
Bupirimate	0.997	12	10	20	30	116	5	100	5	104	3	12	26	0.90
Buprofezin	0.997	9	10	17	23	90	16	90	12	88	8	10	26	0.81
Butocarboxim sulfoxide	0.997	11	10	17	23	107	9	118	8	102	7	7	33	1.06
Cadusafos	0.997	11	10	15	20	110	11	94	8	95	5	6	30	0.85
Carbaryl	0.997	11	10	17	23	105	10	94	5	89	4	9	28	0.85
Carbendazim	0.997	12	10	20	30	118	5	122	5	113	4	11	30	1.10
Carbofuran	0.998	12	10	17	23	116	4	90	4	92	3	8	26	0.81
Carbosulfan	0.997	9	10	21	33	91	10	106	6	93	6	15	32	0.96
Carboxin	0.998	11	10	18	26	113	6	90	4	89	5	12	28	0.81
Clofentezine	0.998	9	10	17	23	88	13	98	10	93	8	10	26	0.88
Chloridazon	0.998	10	10	18	26	102	11	114	9	97	7	11	30	1.03
Chlorfenvinphos	0.998	11	10	18	26	112	8	96	5	100	3	10	30	0.86
Chlorfluazuron	0.998	11	10	21	33	107	10	114	5	108	6	14	30	1.03
Chloroxuron	0.997	11	10	18	26	108	13	102	5	104	5	10	36	0.92
Chlorpyrifos	0.997	11	10	17	23	108	9	102	6	107	6	7	32	0.92
Chlorsulfuron	0.997	10	10	21	33	96	10	92	5	88	5	15	30	0.83
Chlorthiamid	0.997	9	10	21	33	92	17	104	13	114	9	13	32	0.94
Cinidon-ethyl	0.997	11	10	17	23	105	7	100	6	110	5	8	32	0.90
Cyazofamid	0.997	11	10	18	26	112	7	94	7	93	4	10	28	0.85
Cyclanilide	0.997	12	10	21	33	115	5	104	5	113	5	13	30	0.94
Cycloate	0.998	11	10	17	23	106	12	96	11	88	5	9	28	0.86
Cymoxanil	0.998	11	10	17	23	109	7	112	6	103	6	8	32	1.01
Cyproconazole	0.998	10	10	18	26	96	10	92	6	98	4	12	28	0.83
Cyprodinil	0.998	9	10	20	30	91	16	98	9	110	9	11	30	0.88
Demeton-S-methyl	0.998	10	10	20	30	96	16	98	11	101	4	11	28	0.88
Demeton-S-methylsulfoxide	0.998	10	10	13	17	100	8	101	7	99	6	11	35	0.91
Desmedipham	0.998	10	10	13	17	99	8	98	8	87	7	14	0	0.88
Diallate	0.998	10	10	20	30	100	13	120	12	110	6	14	26	1.08
Diazinon	0.998	10	10	12	13	95	9	92	6	100	5	12	32	0.83
Dichlofluanid	0.998	10	10	17	23	103	9	104	8	100	8	12	26	0.94
Dichlorprop	0.998	9	10	18	26	88	5	88	4	95	3	8	0	0.82
Dichlorvos	0.998	9	10	15	20	93	14	118	11	109	11	16	35	1.06
Difenoconazole	0.998	9	10	15	20	92	12	100	9	94	4	11	28	0.90
Dimethenamid (sum)	0.998	11	10	17	23	112	13	98	9	87	10	10	28	0.94
Dimethoate	0.998	11	10	17	23	113	8	104	7	100	6	9	26	0.94
Dimethomorph	0.998	9	10	13	17	91	12	104	8	97	5	14	28	0.94
Dimoxystrobin	0.998	10	10	15	20	101	8	116	5	97	5	12	30	1.05
Diniconazole	0.998	10	10	13	17	97	19	96	15	87	9	10	36	0.86
Dinobuton	0.998	10	10	17	23	104	10	78	6	92	6	11	32	0.82
Dinocap (sum)	0.998	12	10	13	17	115	7	96	5	90	4	14	30	0.86
Dinoterb	0.997	12	10	18	26	115	7	100	4	98	4	9	28	0.90
Diphenylamine	0.999	12	10	13	17	115	7	108	5	90	4	14	30	0.97
Disulfoton-sulfoxide	0.997	11	10	13	17	109	10	86	6	94	5	13	30	0.82
Dithianon	0.997	11	10	13	17	106	6	108	5	102	3	6	26	0.97
Diuron	0.999	11	10	20	30	109	9	94	7	101	3	10	26	0.85
Epoxiconazole	0.997	11	10	12	13	105	8	98	4	93	4	11	28	0.88
EPTC	0.997	11	10	18	26	113	7	112	4	96	4	10	28	1.01
Ethiofencarb	0.997	12	10	18	26	115	7	96	5	96	3	9	26	0.86
Ethion	0.998	10	10	15	20	102	10	100	8	98	8	12	22	0.90
Ethirimol	0.998	10	10	15	20	98	15	96	9	111	8	13	33	0.86
Ethofumesate	0.998	11	10	17	23	105	11	96	10	100	7	13	28	0.86
Etoxazole	0.998	11	10	17	23	114	8	94	5	101	4	11	28	0.85
Ethoxyquin	0.998	10	10	13	17	100	13	96	11	98	4	11	30	0.86
Famoxadone	0.998	11	10	15	20	112	12	114	5	117	5	12	32	1.03
Fenamidone	0.998	11	10	13	17	108	9	84	8	91	6	10	26	0.82
Fenamiphos	0.998	11	10	17	23	107	5	106	4	109	3	5	32	0.96
Fenarimol	0.998	10	10	13	17	101	11	99	9	95	8	11	23	0.89
Fenazaquin	0.997	11	10	18	26	113	8	128	7	113	8	10	28	1.15
Fenbuconazole	0.997	9	10	13	17	86	16	94	12	97	4	8	32	0.85
Fenhexamid	0.997	11	10	18	26	114	7	80	6	84	5	13	28	0.82
Fenitrothion	0.997	10	10	17	23	99	12	98	11	94	9	11	24	0.88
Fenoxycarb	0.997	9	10	13	17	85	16	100	14	103	8	9	35	0.90
Fenpropathrin	0.997	10	10	18	26	100	12	100	10	99	9	7	25	0.90
Fenthion	0.997	10	10	23	36	95	12	112	11	108	7	12	33	1.01
Flazasulfuron	0.998	10	10	30	49	95	10	118	8	116	7	15	30	1.06
Fludioxonil	0.998	12	10	23	36	115	7	88	5	100	5	13	25	0.82
Fluazifop-P-butyl	0.998	12	10	28	46	118	8	102	6	96	5	11	32	0.92
Flufenacet	0.998	10	10	25	40	103	10	96	9	100	7	13	28	0.86
Flufenoxuron	0.998	10	10	26	43	95	8	100	8	105	4	11	32	0.90
Flurochloridone	0.998	10	10	18	26	100	10	102	9	112	6	7	28	0.92
Flurtamone	0.998	10	10	20	30	103	6	90	4	89	4	10	32	0.81
Flusilazole	0.998	10	10	23	36	104	8	92	6	99	6	11	32	0.83
Flutolanil	0.998	12	10	18	26	115	6	114	7	99	7	10	26	1.03
Foramsulfuron	0.998	11	10	25	40	105	4	86	3	99	4	14	26	0.82
Fosthiazate	0.997	8	10	20	30	82	13	94	5	94	3	13	30	0.85
Furathiocarb	0.997	12	10	21	33	115	5	96	5	90	5	12	33	0.86
Heptenophos	0.997	10	10	31	53	99	15	94	12	90	7	11	28	0.85
Hexythiazox	0.997	10	10	25	40	103	6	100	4	98	5	9	32	0.90
Imazalil	0.997	10	10	28	46	95	12	100	11	88	10	9	28	0.90
Imazamox	0.997	8	10	18	26	84	11	100	9	92	6	12	26	0.90
Imazaquin	0.997	11	10	31	53	112	9	90	5	86	3	12	0	0.81
Imazosulfuron	0.997	10	10	25	40	98	12	95	11	96	8	11	36	0.86
Imidacloprid	0.999	9	10	30	49	87	12	102	9	109	6	15	26	0.92
Indoxacarb	0.999	10	10	23	36	99	15	100	6	93	8	16	30	0.90
Ioxynil	0.999	10	10	20	30	99	4	84	4	81	5	8	30	0.81
Iprovalicarb	0.999	10	10	20	30	97	12	110	8	104	7	15	32	0.99
Isazofos	0.999	12	10	21	33	115	7	94	5	89	11	13	26	0.85
Isoproturon	0.999	12	10	20	30	115	8	90	6	97	6	11	38	0.81
Isoxaben	0.997	11	10	26	43	112	5	110	3	96	5	8	30	0.99
Lufenuron	0.997	10	10	26	43	99	4	98	4	94	4	13	30	0.88
Malathion	0.999	12	10	23	36	115	7	104	5	89	6	13	33	0.94
MCPA	0.997	9	10	25	40	93	9	102	5	103	7	11	28	0.92
Mecarbam	0.998	11	10	31	53	105	16	106	14	94	7	11	30	0.96
MCPP	0.998	10	10	28	46	103	12	84	9	97	9	12	41	0.82
Metalaxyl-M	0.998	10	10	23	36	97	7	122	4	101	5	16	26	1.10
Mepanipyrim	0.997	10	10	31	53	102	16	92	13	107	11	16	33	0.83
Mesosulfuron-methyl	0.997	8	10	28	46	82	15	104	5	93	6	12	35	0.94
Metazachlor	0.997	11	10	23	36	113	5	100	5	93	6	12	16	0.90
S-Metolachlor	0.999	9	10	18	26	93	8	92	7	97	7	11	27	0.83
Metosulam	0.999	11	10	30	49	110	9	94	8	88	8	14	32	0.85
Metribuzin	0.999	11	10	21	33	105	11	72	9	91	6	15	26	0.82
Monocrotophos	0.997	12	10	25	40	115	4	100	3	88	3	14	32	0.90
Monolinuron	0.997	10	10	28	46	104	17	92	14	90	8	13	28	0.83
Monuron	0.997	11	10	31	53	114	8	102	4	94	5	13	35	0.92
Omethoate	0.997	11	10	28	46	105	9	94	9	91	8	13	39	0.85
Oxadiargyl	0.997	11	10	23	36	105	15	110	11	90	11	14	30	0.99
Oxadiazon	0.997	10	10	30	49	96	8	112	5	112	5	6	32	1.01
Oxadixyl	0.997	11	10	21	33	109	12	90	10	88	6	8	35	0.81
Oxamyl	0.997	10	10	30	49	103	14	96	12	89	8	11	33	0.86
Oxasulfuron	0.997	11	10	33	56	108	8	86	8	87	7	12	28	0.82
Oxycarboxin	0.997	12	10	28	46	115	7	112	4	97	6	12	28	1.01
Oxyfluorfen	0.997	9	10	30	49	92	8	101	7	103	6	11	28	0.91
Penconazole	0.997	11	10	23	36	112	7	102	4	96	4	9	28	0.92
Pendimethalin	0.997	11	10	20	30	113	8	108	5	108	4	6	28	0.97
Pethoxamid	0.997	11	10	28	46	108	9	76	4	81	5	14	32	0.83
Phosalone	0.998	10	10	23	36	96	11	112	4	112	5	8	36	1.01
Phenmedipham	0.998	10	10	36	62	95	14	92	13	88	6	11	30	0.83
Phenthoate	0.997	12	10	23	36	115	7	94	5	89	6	12	28	0.85
Phosmet	0.997	10	10	21	33	100	11	94	10	92	9	13	33	0.85
Phosphamidon	0.997	10	10	21	33	99	8	108	7	95	7	10	30	0.97
Picloram	0.997	11	10	21	33	105	12	96	8	91	7	12	36	0.86
Picolinafen	0.997	10	10	25	40	100	12	114	9	104	9	12	25	1.03
Pirimicarb	0.997	12	10	23	36	119	8	94	2	97	3	10	30	0.85
Pirimiphos-methyl	0.997	12	10	23	36	115	7	108	5	94	5	12	30	0.97
Prochloraz	0.999	12	10	21	33	115	5	112	5	94	5	18	30	1.01
Profenofos	0.999	12	10	21	33	115	7	104	5	96	5	8	30	0.94
Prometryn	0.999	11	10	21	33	109	7	94	5	99	6	9	36	0.85
Propamocarb	0.998	10	10	25	40	100	13	102	10	89	9	11	30	0.92
Propanil	0.999	12	10	30	49	115	7	102	5	99	6	12	28	0.92
Propargite	0.999	10	10	26	43	102	14	112	10	113	4	8	30	1.01
Propham	0.999	10	10	21	33	102	13	100	6	96	5	10	28	0.90
Propiconazole	0.999	10	10	30	49	98	6	122	4	111	6	14	30	1.10
Propyzamide	0.999	8	10	28	46	84	10	96	9	95	5	9	33	0.86
Pymetrozine	0.997	10	10	23	36	101	10	96	8	90	7	11	28	0.86
Pyraclostrobin	0.997	12	10	21	33	115	7	130	5	119	4	15	26	1.17
Pyridaben	0.997	9	10	20	30	89	16	80	12	81	4	11	36	0.82
Pyridaphenthion	0.997	10	10	23	36	99	11	120	9	99	10	13	26	1.08
Pyridate	0.998	10	10	31	53	98	5	106	4	98	3	8	32	0.96
Pyriproxyfen	0.997	10	10	21	33	101	11	94	6	86	5	13	22	0.85
Quinalphos	0.997	10	10	23	36	101	5	76	6	90	5	14	23	0.82
Quinoxyfen	0.999	10	10	30	49	99	5	98	1	100	2	8	23	0.88
Quizalofop-P-ethyl	0.997	12	10	30	49	115	7	82	5	85	5	14	26	0.84
Rimsulfuron	0.998	9	10	26	43	88	13	104	3	100	2	14	26	0.94
Simazine	0.997	11	10	23	36	106	16	96	8	99	3	11	23	0.86
Spiroxamine	0.999	8	10	21	33	83	14	84	9	82	2	14	32	0.82
Sulfosulfuron	0.997	8	10	21	33	82	12	110	6	111	6	19	30	0.99
Tebuconazole	0.999	10	10	28	46	99	10	94	6	99	5	9	25	0.85
TEPP	0.997	9	10	23	36	90	7	107	7	105	6	11	29	0.96
Terbutryn	0.997	12	10	26	43	115	8	96	5	102	3	11	28	0.86
Terbuthylazine	0.997	10	10	20	30	104	12	118	5	116	4	6	26	1.06
Tetrachlorvinphos	0.997	11	10	21	33	113	11	78	8	90	4	14	28	0.90
Thiabendazole	0.997	12	10	21	33	118	6	98	4	96	5	13	30	0.88
Thiacloprid	0.997	11	10	18	26	106	13	84	12	89	5	8	35	0.82
Thiamethoxam	0.997	10	10	33	56	104	19	118	11	113	8	11	32	1.06
Thifensulfuron-methyl	0.997	9	10	20	30	91	16	98	8	92	9	15	30	0.88
Thiodicarb	0.997	12	10	31	53	115	7	102	5	97	6	9	35	0.92
Thiophanate-methyl	0.997	11	10	20	30	114	9	112	6	95	5	13	32	1.01
Triadimefon	0.997	10	10	28	46	104	7	106	6	98	5	8	32	0.96
Triadimenol	0.997	11	10	25	40	107	14	106	6	92	7	9	26	0.96
Triallate	0.998	11	10	23	36	106	9	114	3	111	4	12	30	1.03
Triasulfuron	0.998	11	10	20	30	111	8	76	5	91	6	14	30	0.82
Triazophos	0.998	12	10	30	49	115	7	96	5	87	5	9	30	0.86
Tribenuron-methyl	0.998	10	10	35	59	99	9	103	8	101	5	11	30	0.93
Tributylphosphate	0.997	10	10	17	23	104	14	88	12	89	5	8	35	0.81
Trichlorfon	0.998	10	10	25	40	98	10	104	3	100	2	14	27	0.94
Tridemorph	0.997	10	10	21	33	98	17	90	13	100	5	11	30	0.81
Trifloxystrobin	0.997	12	10	25	40	115	7	124	5	114	5	14	30	1.12
Triflumizole	0.997	11	10	20	30	113	14	92	5	99	5	8	28	0.83
Triticonazole	0.998	10	10	17	23	100	11	106	10	98	7	7	32	0.96
Vamidothion	0.997	9	10	21	33	93	13	118	6	102	7	16	28	1.06
Zoxamide	0.997	12	10	23	36	116	7	72	4	85	3	15	25	0.82
Ciprofloxacin	0.997	7	100	124	148	74	25	61	16	97	13	9	38	0.81
Clindamycin	0.997	11	10	12	14	110	23	106	15	109	10	12	34	0.96
Chlortetracycline	0.999	10	100	108	116	101	7	93	7	99	6	9	14	0.84
Danofloxacin	0.999	10	30	40	48	104	14	87	12	88	8	9	24	0.82
Difloxacin	0.999	9	10	11	13	87	8	100	7	96	6	10	15	0.90
Doxycycline hydrate	0.999	10	100	110	119	104	14	93	11	96	8	10	23	0.84
Flumequine	0.998	9	50	54	58	88	12	105	11	93	10	11	22	0.95
Josamycin	0.998	9	10	11	13	88	12	104	11	97	9	12	22	0.94
Lomefloxacin	0.999	10	10	12	13	99	13	89	11	99	8	10	22	0.80
Orbifloxacin	0.999	9	10	11	12	93	14	107	8	94	7	11	20	0.96
Oxolinic acid	0.999	11	10	12	15	110	12	102	10	102	6	15	20	0.92
Oxytetracycline	0.999	11	100	111	122	114	15	104	12	95	6	10	23	0.94
Rifampicin	0.997	10	10	11	13	98	9	85	8	98	5	9	15	0.81
Sarafloxacin	0.997	10	10	12	14	101	10	97	8	102	5	11	16	0.87
Sulfachloropyridazine	0.997	9	100	116	132	88	13	106	12	80	9	10	24	0.96
Sulfaquinoxaline	0.998	10	100	111	122	97	15	99	10	78	9	9	24	0.89
Sulfadiazine	0.997	8	100	108	116	85	12	98	9	98	5	10	18	0.88
Sulfamerazine	0.999	8	100	111	122	85	12	104	8	75	7	9	19	0.94
Sulfathiazole	0.999	9	100	113	126	85	11	109	10	81	10	17	21	0.98
Sulfamethazine	0.999	8	100	114	129	84	13	104	9	104	8	11	21	0.94
Sulfadoxine	0.999	9	100	114	129	88	16	95	12	95	6	10	24	0.86
Sulfapyridine	0.997	8	100	119	138	83	11	108	10	96	8	10	20	0.97
Sulfaclozine	0.998	10	100	111	122	99	10	92	8	98	5	9	16	0.83
Sulfamethoxazole	0.998	10	100	116	132	102	9	95	7	87	5	9	15	0.86
Tilmicosin	0.999	10	50	56	62	98	12	104	9	105	4	10	18	0.94
Tetracycline	0.999	10	100	119	138	100	18	84	8	96	5	9	23	0.82

## References

[1] Zhang Y., Li X., Liu X. (2015). Multi-class, multi-residue analysis of trace veterinary drugs in milk by rapid screening and quantification using ultra-performance liquid chromatography–quadrupole time-of-flight mass spectrometry. *Journal of Dairy Science*.

[2] Rekha, Naik S. N., Prasad R. (2006). Pesticide residue in organic and conventional food-risk analysis. *Journal of Chemical Health and Safety*.

[3] European Union (2010). Commission Regulation No 37/2010 of 22nd December 2009 on pharmacologically active substances and their classification regarding maximum residue limits in foodstuffs of animal origin. *Official Journal of the European Communities International*.

[4] Aguilera-Luiz M. M., Vidal J. L. M., Romero-González R., Frenich A. G. (2008). Multi-residue determination of veterinary drugs in milk by ultra-high-pressure liquid chromatography-tandem mass spectrometry. *Journal of Chromatography A*.

[5] Turnipseed S. B., Andersen W. C., Karbiwnyk C. M., Madson M. R., Miller K. E. (2008). Multi-class, multi-residue liquid chromatography/tandem mass spectrometry screening and confirmation methods for drug residues in milk. *Rapid Communications in Mass Spectrometry*.

[6] Aguilera-Luiz M. M., Plaza-Bolaños P., Romero-González R., Vidal J. L. M., Frenich A. G. (2011). Comparison of the efficiency of different extraction methods for the simultaneous determination of mycotoxins and pesticides in milk samples by ultra high-performance liquid chromatography-tandem mass spectrometry. *Analytical and Bioanalytical Chemistry*.

[7] Kaufmann A., Butcher P., Maden K., Walker S., Widmer M. (2011). Quantification of anthelmintic drug residues in milk and muscle tissues by liquid chromatography coupled to orbitrap and liquid chromatography coupled to tandem mass spectrometry. *Talanta*.

[8] Stolker A. A. M., Rutgers P., Oosterink E. (2008). Comprehensive screening and quantification of veterinary drugs in milk using UPLC-ToF-MS. *Analytical and Bioanalytical Chemistry*.

[9] Gaugain-Juhel M., Delépine B., Gautier S. (2009). Validation of a liquid chromatography-tandem mass spectrometry screening method to monitor 58 antibiotics in milk: a qualitative approach. *Food Additives and Contaminants—Part A*.

[10] Tang Y.-Y., Lu H.-F., Lin H.-Y., Shih Y.-C., Hwang D.-F. (2012). Multiclass analysis of 23 veterinary drugs in milk by ultraperformance liquid chromatography-electrospray tandem mass spectrometry. *Journal of Chromatography B*.

[11] Zhan J., Yu X.-J., Zhong Y.-Y. (2012). Generic and rapid determination of veterinary drug residues and other contaminants in raw milk by ultra performance liquid chromatography–tandem mass spectrometry. *Journal of Chromatography B*.

[12] Turnipseed S. B., Storey J. M., Clark S. B., Miller K. E. (2011). Analysis of veterinary drugs and metabolites in milk using quadrupole time-of-flight liquid chromatography-mass spectrometry. *Journal of Agricultural and Food Chemistry*.

[13] Romero-González R., Aguilera-Luiz M. M., Plaza-Bolaños P., Frenich A. G., Vidal J. L. M. (2011). Food contaminant analysis at high resolution mass spectrometry: application for the determination of veterinary drugs in milk. *Journal of Chromatography A*.

[14] Garrido Frenich A., Aguilera-Luiz M. D. M., Martínez Vidal J. L., Romero-González R. (2010). Comparison of several extraction techniques for multiclass analysis of veterinary drugs in eggs using ultra-high pressure liquid chromatography–tandem mass spectrometry. *Analytica Chimica Acta*.

[15] Lehotay S., O'Neil M., Tully J. (2007). Determination of pesticide residues in foods by acetonitrile extraction and partitioning with magnesium sulfate: collaborative study. *Journal of the European Communities International*.

[16] European Commission Decision (2002). 2002/657/EC, implementing Council Directive 96/23/EC, concerning the performance of analytical methods and interpretation of the results. *Official Journal of the European Communities International*.

[17] http://ec.europa.eu/food/plant/docs/plant_pesticides_mrl_guidelines_wrkdoc_11945_en.pdf.

[18] Kaufmann A., Butcher P., Maden K., Walker S., Widmer M. (2014). Multi-residue quantification of veterinary drugs in milk with a novel extraction and cleanup technique: salting out supported liquid extraction (SOSLE). *Analytica Chimica Acta*.

[19] Lehotay S. J. (2007). Determination of pesticides residues in food by acetonitrile extraction and partitioning with magnesium sulfate: Collaborative study. *The Journal of AOAC International*.

[20] Gao F., Zhao Y., Shao B., Zhang J. (2012). Determination of residues of pesticides and veterinary drugs in milk by ultra performance liquid choromatography coupled with quadrupole time of fligt mass spectrometry. *Chinese Journal of Chromatography*.

